# Health-related quality of life is a significant prognostic factor for recurrence and overall survival in patients with colon cancer

**DOI:** 10.1186/s12885-025-14254-1

**Published:** 2025-06-10

**Authors:** Catarina Tiselius, Fanny Johansen, Andreas Rosenblad, Kenneth Smedh

**Affiliations:** 1 Institution for Surgical Sciences, Uppsala University, Uppsala, Sweden; 2 Centre for Clinical research, Västmanlands sjukhus Västerås, Västerås, Sweden; 3 Regional Cancer Centre Stockholm-Gotland, Stockholm, Sweden; 4 Department of Statistics, Uppsala University, Uppsala, Sweden; 5 Division of Clinical Diabetology and Metabolism, Department of Medical Science, Uppsala University, Uppsala, Sweden

**Keywords:** Colon cancer, Health-related quality of life, Recurrence, Survival

## Abstract

**Background:**

Health-related quality of life (HRQoL) is associated with survival in patients with cancer; however, there are few studies on the risk of cancer recurrence. We investigated whether HRQoL can predict disease-free and overall survival (DFS/OS) in patients with non-metastatic colon cancer.

**Methods:**

This population-based prospective study investigated patients diagnosed with colon cancer between 2012 and 2016. The 30-item European Organisation for Research and Treatment of Cancer Core Quality of Life Questionnaire (EORTC QLQ-C30) was used to measure HRQoL at diagnosis. Cox proportional hazard regression analyses were used to analyse the association between QLQ-C30 scores and DFS/OS.

**Results:**

Of the 323 patients with non-metastatic colorectal cancer, *n* = 41 (12.7%) were diagnosed with recurrence during mean (standard deviation) DFS and OS follow-up times of 5.9 (2.9) and 6.2 (2.7) years, respectively. Cox regression analysis of HRQoL, adjusted for important clinical and demographic variables, showed that a higher global health status was significantly associated with an improved DFS (hazard ratio [HR] 0.86 per 10 points; 95% confidence interval [CI] 0.79–0.94; *P* < 0.001) as well as OS (HR 0.88 per 10 points; 99% CI 0.80–0.96; *P* = 0.003).

**Conclusions:**

These results demonstrate that HRQoL can predict both DFS and OS in patients with non-metastatic colon cancer. HRQoL should be considered an additional tool in non-metastatic cancer for assessing patients at risk of metastatic disease.

**Trial registration:**

ClinicalTrials.gov (NCT 03910894).

## Background

Colon cancer is the third most common cancer worldwide. Its prevalence and incidence are increasing; therefore, management of this disease is highly important [1]. About 22 to 35% of patients have metastases at diagnosis [2, 3] and about 36% experience recurrence [4, 5]. Cancer recurrence and metastasis are the most significant clinical issues that determine patient outcomes. Not only are surgical and oncological treatment of high importance, but health-related quality of life (HRQoL) is also very important for patients with cancer. The prognostic value of tumour- and surgery-related factors is well studied, but physical factors such as comorbidity are less understood [6]. However, these factors cannot fully predict the high recurrence risk and overall survival (OS) [7]. Earlier studies have indicated that stress-related psychological factors are associated with an increased incidence and poorer survival in some cancer types [8]. A recent review showed a moderate correlation between psychological stress and cancer recurrence in patients with breast cancer [9]. A study from Japan showed a higher relative risk of cancer-related death in patients with higher stress levels [4]. In patients with non-metastatic colon cancer, distress has been shown to have a negative impact on recurrence [10]. A healthy diet and lifestyle have been shown to have the opposite effect and may significantly decrease the risk of colon cancer recurrence and improve survival [7]. There is increasing evidence that HRQoL, measured with the 30-item European Organisation for Research and Treatment of Cancer Core Quality of Life Questionnaire (EORTC QLQ-C30), can also predict survival [11–13]; however, whether HRQoL can predict the recurrence of metastases in colon cancer remains unclear.

### Aim

This study aimed to examine whether HRQoL assessed using the EORTC QLQ-C30 instrument could predict the risk of cancer recurrence, measured as disease-free survival (DFS) and OS, in patients with non-metastatic colon cancer.

## Methods

### Study population

**Fig. 1:**
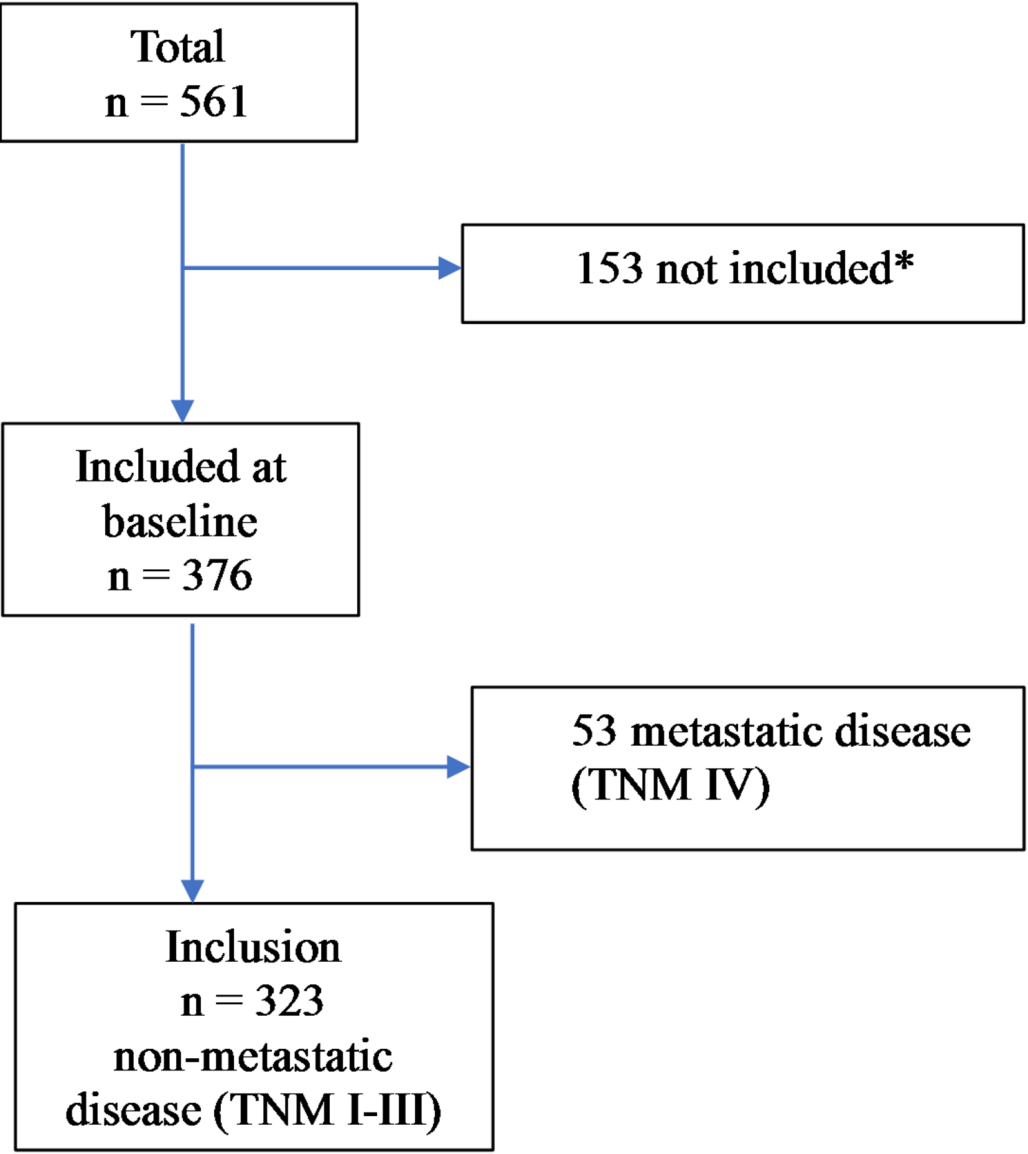
Flow chart of the number of patients diagnosed with colon cancer between March 2012 and September 2016 in Västmanland County, Sweden (* reference 14)

A detailed description of the data collection process has been given in a previously published study [14]. The study population consisted of 561 patients diagnosed with colon cancer between March 2012 and September 2016 in Västmanland County, Sweden (Fig. 1). In brief, after the patients provided written informed consent, they were enrolled in the study and completed the baseline questionnaire, including the EORTC QLQ-C30 instrument, within 1 month of diagnosis and before the start of treatment. Follow-up questionnaires were answered at follow-ups taking place 6 months, 1 year, and 3 years after baseline. Patients who did not consent to be included in the study or were unable to understand or answer the questionnaire were excluded. They were followed-up until June 4, 2022. Additional data on patient characteristics, including age, sex, American Society of Anesthesiologists (ASA) classification, body mass index (BMI), tumour location, preoperative tumour stage, and surgery were collected from medical reports. Patients were followed-up according to the Swedish National Guidelines, undergoing computer tomography after 1 and 3 years and endoscopic control with colonoscopy after 3 years and subsequently every 5 years until the patient turned 75 years old. Patients with tumour-node-metastasis (TNM) stage III received adjuvant chemotherapy if they were not too old, had severe comorbidity, or low World Health Organization performance status.

### Outcome and measures

The primary outcome measures were local recurrence and appearance of distant metastases in patients with non-metastatic (TNM stage I-III) disease. Local recurrence was confirmed by a biopsy sample and/or positive imaging. Distant metastases were defined as recurrent disease in the peritoneum, liver, or outside the abdomen. The date of first site of recurrence was calculated, and DFS was defined as time to first event (local recurrence, distant metastasis, or death), with an individual considered censored at the date of follow-up (June 4, 2022) if it had not yet experienced any of these events at this date. The secondary outcome measure was OS, defined as the time from diagnosis to death from any cause. In this case, an individual was considered censored at the date of follow-up (June 4, 2022) if it was still alive at this date.

### HRQoL questionnaire

HRQoL was assessed using the well-validated generic EORTC QLQ-C30 questionnaire [15]. It consists of a global health status scale, five functional scales (physical, role, emotional, cognitive, and social), three symptom scales (fatigue, nausea/vomiting, and pain), and six single items (dyspnoea, insomnia, appetite loss, constipation, diarrhoea, and financial difficulties). All scales and items have scores ranging from 0 to 100 points. A high score for the global QoL or functional scale is interpreted as representing a high level of QoL or functioning, while a high score for symptoms scales/items is interpreted as representing more severe symptomatology.

### Statistical analyses

Categorical data are given as frequencies and percentages, *n* (%), while continuous data are given as means with accompanying standard deviations (SDs). Kaplan–Meier plots are used for graphically describing the univariate survival probability over time for categorical data, with differences between groups tested using associated log-rank tests. Unadjusted (univariable) and adjusted (multivariable) Cox proportional hazards regression models were used to estimate how HRQoL, age, sex, BMI, comorbidity (measured with ASA status) and TNM stage I-III were associated with DFS and OS. The results of the Cox regression models are given as hazard ratios (HRs) with accompanying 95% confidence intervals (CIs), with the association between HRQoL and DFS/OS estimated using separate models for each of.

Global health status (GHS), the five functional scales, and nine symptom scales/items. For ease of interpretation, the HRs and 95% CIs for the HRQoL variables are given per 10 points of the 0–100 points range. SPSS 28.0 (IBM, Armonk, NY, USA) and R 4.4.2 (R foundation for Statistical Computing, Vienna, Austria) were used for the statistical analysis, with P-values < 0.05 considered statistically significant. In line with the arguments by Rothman (1990) that adjustments for multiple comparisons should be avoided when analysing empirical data from actual observations, since these are not random numbers but should be considered as mirroring underlying natural processes, no adjustments for multiple comparisons were performed [16].

## Results

A total of 376 (67.0%) patients answered the HRQoL questionnaire, 323 (85.9%) of whom were diagnosed with non-metastatic colon cancer (TNM stage I–III), as shown in Fig. 1. Clinical and demographic characteristics of the 376 patients answering the HRQoL questionnaire are given in Table 1, with data on patients with metastatic (TNM stage IV) disease included for completeness. The 323 patients with TNM stage I–III were at a mean (SD) age of 73.6 (10.6) years, with a slight minority (*n* = 156; 48.3%) being males. Most of the participants were at TNM stage II (*n* = 155; 48.0%) or III (*n* = 132; 40.9%), with only one tenth (*n* = 36; 11.1%) being at TNM stage I. The total mean (SD) DFS and OS follow-up times for the 323 patients with TNM stage I–III were 5.9 (2.9) and 6.2 (2.7) years, respectively. During follow-up, 41 (12.7%) of the 323 patients experienced cancer recurrence, with a mean (SD) time to recurrence of 1.5 (1.1) years.

**Table 1:** Clinical and demographic characteristics of the 376 patients answering the HRQoL questionnaire

	TNM I–III			TNM IV
	Total	Disease-free	Recurrence	
Variable	*n* = 323	*n* = 282 (87.3%)	*n* = 41 (12.7%)	*n* = 53
Age (years), mean (SD)	73.6 (10.6)	73.6 (10.5)	73.7 (11.3)	71.5 (12.5)
Male sex, *n* (%)	156 (48.3)	135 (47.9)	21 (51.2)	26 (49.1)
BMI (kg/m^2^), mean (SD)	26.6 (4.9)	26.7 (4.7)	26.2 (6.3)	26.4 (4.4)
ASA status, *n* (%)				
− 1	34 (10.5)	26 (9.2)	8 (19.5)	9 (17.0)
− 2	158 (48.9)	139 (49.3)	19 (46.3)	27 (50.9)
− 3	119 (36.8)	103 (37.6)	13 (31.7)	15 (28.3)
− 4	12 (3.7)	11 (3.9)	1 (2.4)	2 (3.8)
TNM stage, *n* (%)				
− 1	36 (11.1)	35 (12.4)	1 (2.4)	
− 2	155 (48.0)	144 (51.1)	11 (26.8)	
− 3	132 (40.9)	103 (36.5)	29 (70.7)	
− 4				53 (14.1)
Adjuvant chemotherapy, *n* (%)	155 (48.0)	126 (44.7)	29 (70.7)	N/A
Surgery, *n* (%)	306 (94.7)	266 (94.3)	40 (97.6)	37 (69.8)
*Surgically treated patients* ^a^				
Emergency surgery, *n* (%)	48 (15.7)	37 (13.9)	11 (27.5)	16 (43.2)
Stoma				
− Yes, with resection	96 (31.4)	78 (29.3)	18 (45.0)	12 (32.4)
− Yes, without resection	5 (1.6)	5 (1.9)	0 (0.0)	9 (24.3)
− No	205 (67.0)	183 (68.8)	22 (55.0)	16 (43.2)
Postoperative complications, *n* (%)	69 (22.5)	57 (21.4)	12 (30.0)	2 (5.4)

Notes: ASA, American Society of Anesthesiologists; BMI, body mass index; N/A, not applicable; SD, standard deviation; TNM, tumour-node-metastasis. Among the *n* = 40 (12.4%) participants with a recurrence, the recurrence occurred in the liver (*n* = 11; 26.8%), lung (*n* = 9; 22.2%), liver and lung (*n* = 7; 17.1%), or other sites (*n* = 14; 34.1%), including metastases at the abdominal wall, abdomen, lymph nodes, or peritoneal. ^a^ Percentages are calculated based on number of surgically treated patients

An univariable (unadjusted) Cox regression analysis of DFS and OS for patients with tumour stage I-III are shown in Table 2. The results show that older patients, patients with more comorbidity, lower GHS, lower physical-, role- and social functioning, more fatigue, more nausea and vomiting, dyspnoea and appetite loss had significantly worse DFS and OS.

**Table 2:** Results from univariable (unadjusted) Cox regression analyses of demographic, clinical, and HRQoL (per 10 points) variables at baseline (diagnosis) for patients with tumour stage I–III in relation to disease-free survival (DFS) and overall survival (OS)

		Disease-free survival			Overall survival		
Variables	n	Events	HR (95% CI)	P-value	Events	HR (95% CI)	P-value
Age (years)	322	131	1.04 (1.02–1.07)	**< 0.001**	121	1.06 (1.03–1.09)	**< 0.001**
Male sex	323	131	1.23 (0.87–1.74)	0.233	121	1.27 (0.88–1.82)	0.195
BMI (kg/m^2^)	318	128	1.02 (0.98–1.06)	0.359	118	1.02 (0.98–1.06)	0.301
ASA status	323	131			121		
− 1			Ref.			Ref.	
− 2			0.77 (0.39–1.49)	0.428		0.89 (0.43–1.83)	0.748
− 3			1.69 (0.89–3.21)	0.109		2.16 (1.07–4.36)	**0.031**
− 4			4.36 (1.84–10.32)	**< 0.001**		6.81 (2.75–16.86)	**< 0.001**
TNM stage	323	131			121		
− 1			Ref.			Ref.	
− 2			1.06 (0.56–1.99)	0.851		1.04 (0.54–2.01)	0.902
− 3			1.83 (0.98–3.40)	0.054		1.76 (0.92–3.35)	0.085
HRQoL (10 points)							
− Global health status	312	127	0.83 (0.76–0.91)	**< 0.001**	117	0.83 (0.76–0.91)	**< 0.001**
• Functional scales							
− Physical functioning	301	125	0.83 (0.76–0.90)	**< 0.001**	116	0.79 (0.72–0.86)	**< 0.001**
− Role functioning	307	126	0.89 (0.84–0.94)	**< 0.001**	116	0.89 (0.84–0.94)	**< 0.001**
− Emotional functioning	308	125	0.95 (0.08–1.03)	0.226	115	0.97 (0.89–1.06)	0.495
− Cognitive functioning	312	125	0.93 (0.85–1.01)	0.079	115	0.93 (0.85–1.01)	0.074
− Social function	309	124	0.89 (0.83–0.96)	**0.001**	115	0.89 (0.83–0.96)	**0.001**
• Symptom scales/items							
− Fatigue	313	128	1.11 (1.03–1.19)	**0.003**	118	1.12 (1.05–1.21)	**< 0.001**
− Nausea and vomiting	315	128	1.16 (1.06–1.27)	**0.001**	118	1.11 (1.02–1.23)	**0.023**
− Pain	313	127	1.06 (0.99–1.13)	0.056	117	1.05 (0.98–1.12)	0.132
− Dyspnoea	312	126	1.08 (1.02–1.15)	**0.008**	117	1.10 (1.03–1.17)	**0.002**
− Insomnia	314	128	1.00 (0.94–1.07)	0.977	118	0.99 (0.92–1.05)	0.678
− Appetite loss	314	128	1.10 (1.04–1.16)	**< 0.001**	118	1.09 (1.03–1.16)	**< 0.001**
− Constipation	312	126	1.02 (0.95–1.09)	0.551	116	1.01 (0.94–1.09)	0.798
− Diarrhoea	312	127	1.03 (0.97–1.09)	0.254	117	1.04 (0.98–1.11)	0.163
− Financial difficulties	312	126	0.97 (0.89–1.07)	0.562	116	0.94 (0.85–1.05)	0.274

Notes: ASA, American Society of Anesthesiologists; BMI, body mass index; CI, confidence interval; HR, hazard ratio; HRQoL, heath-related quality of life; Ref., reference category; TNM, tumour-node-metastasis. Statistically significant P-values are given in **bold**

Data on multivariable (adjusted) Cox regression analyses of DFS and of OS at baseline (diagnosis) are presented in Table 3. The tables show the analysis of HRQoL in patients with colon cancer with non-metastatic disease (TNM stage I–III), adjusted for age, sex, BMI, ASA status and TNM stage. At diagnosis, a lower GHS, lower physical, role, and social functioning and more fatigue were associated with significantly worse DFS and OS. In addition, a lower emotional functioning, more nausea and vomiting, and pain were associated with significantly worse DFS, and more diarrhoea was associated with a significantly worse OS.

**Table 3:** Results from multivariable (adjusted) Cox regression analyses of HRQoL (per 10 points) variables at baseline (diagnosis) for patients with tumour stage I–III in relation to disease-free survival (DFS) and overall survival (OS), adjusted for age (years), sex, BMI (kg/m^2^), ASA status, and TNM stage

		Disease-free survival			Overall survival		
Variables	n	Events	HR (95% CI)	P-value	Events	HR (95% CI)	P-value
HRQoL (10 points)							
− Global health status	306	124	0.86 (0.79–0.94)	**< 0.001**	114	0.88 (0.80–0.96)	**0.003**
• Functional scales							
− Physical functioning	295	122	0.88 (0.80–0.96)	**0.003**	113	0.84 (0.77–0.93)	**< 0.001**
− Role functioning	301	123	0.92 (0.87–0.97)	**0.001**	113	0.92 (0.87–0.98)	**0.002**
− Emotional functioning	303	123	0.92 (0.84-1.00)	**0.032**	113	0.93 (0.85–1.02)	0.092
− Cognitive functioning	306	122	0.93 (0.85–1.01)	0.079	112	0.92 (0.84–1.01)	0.072
− Social function	303	121	0.89 (0.83–0.95)	**< 0.001**	112	0.89 (0.82–0.95)	**< 0.001**
• Symptom scales/items							
− Fatigue	307	125	1.08 (1.00-1.16)	**0.035**	115	1.09 (1.01–1.17)	**0.026**
− Nausea and vomiting	309	125	1.15 (1.05–1.26)	**0.001**	115	1.09 (0.99–1.19)	0.072
− Pain	307	124	1.08 (1.00-1.15)	**0.025**	114	1.06 (0.98–1.13)	0.102
− Dyspnoea	306	123	1.02 (0.96–1.09)	0.454	114	1.02 (0.96–1.10)	0.457
− Insomnia	308	125	1.00 (0.94–1.06)	0.922	115	0.98 (0.91–1.04)	0.465
− Appetite loss	308	125	1.10 (1.03–1.16)	**< 0.001**	115	1.08 (1.02–1.15)	**0.006**
− Constipation	306	123	1.04 (0.96–1.11)	0.318	113	1.01 (0.94–1.09)	0.756
− Diarrhoea	306	124	1.05 (0.99–1.12)	0.061	114	1.07 (1.01–1.14)	**0.017**
− Financial difficulties	306	123	1.00 (0.90–1.11)	0.977	113	0.97 (0.86–1.08)	0.535

Notes: ASA, American Society of Anesthesiologists; BMI, body mass index; CI, confidence interval; HR, hazard ratio; HRQoL, heath-related quality of life; TNM, tumour-node-metastasis. Statistically significant P-values are given in **bold**

**Fig. 2:**
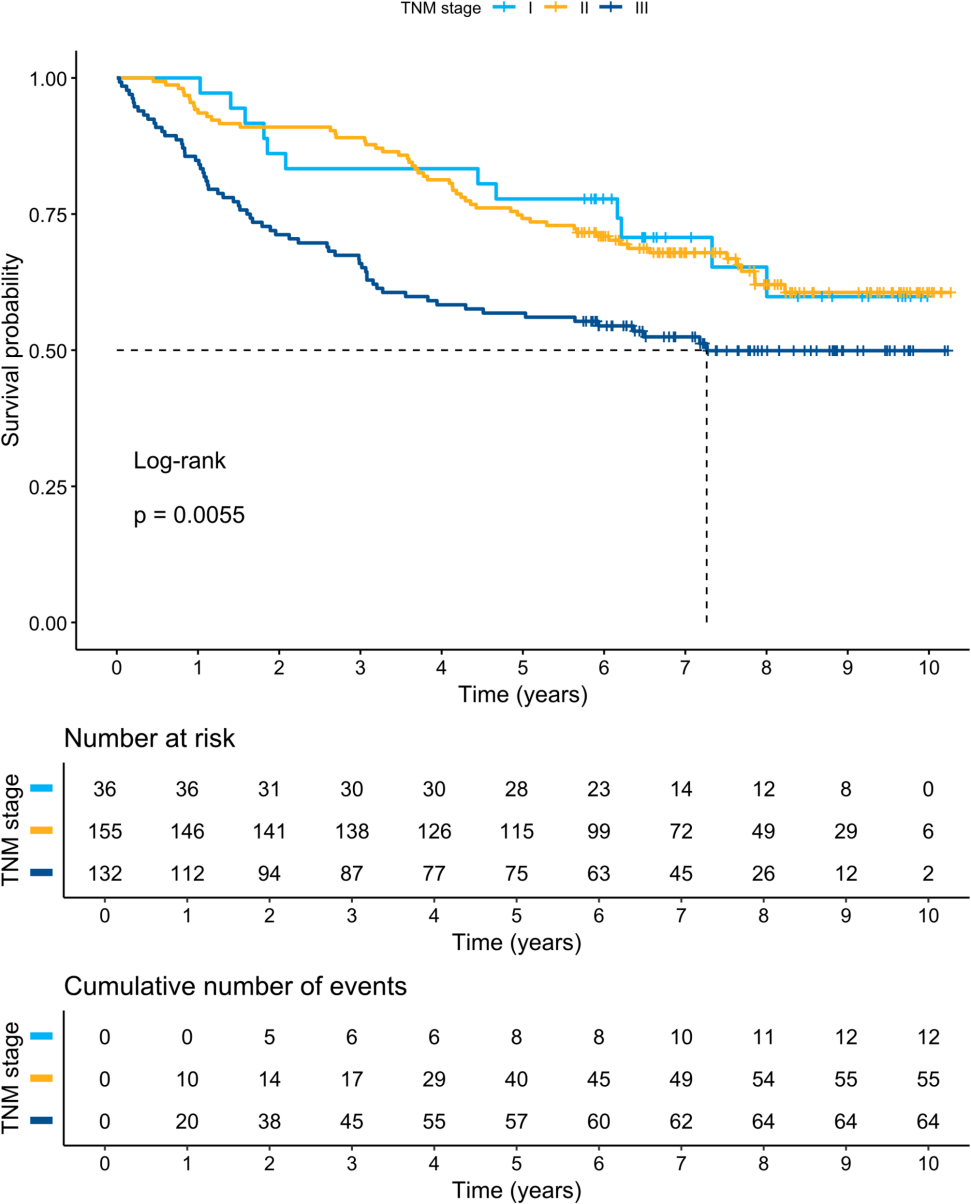
Kaplan-Meier curves for TNM stage in relation to disease-free survival (DFS)

**Fig. 3:**
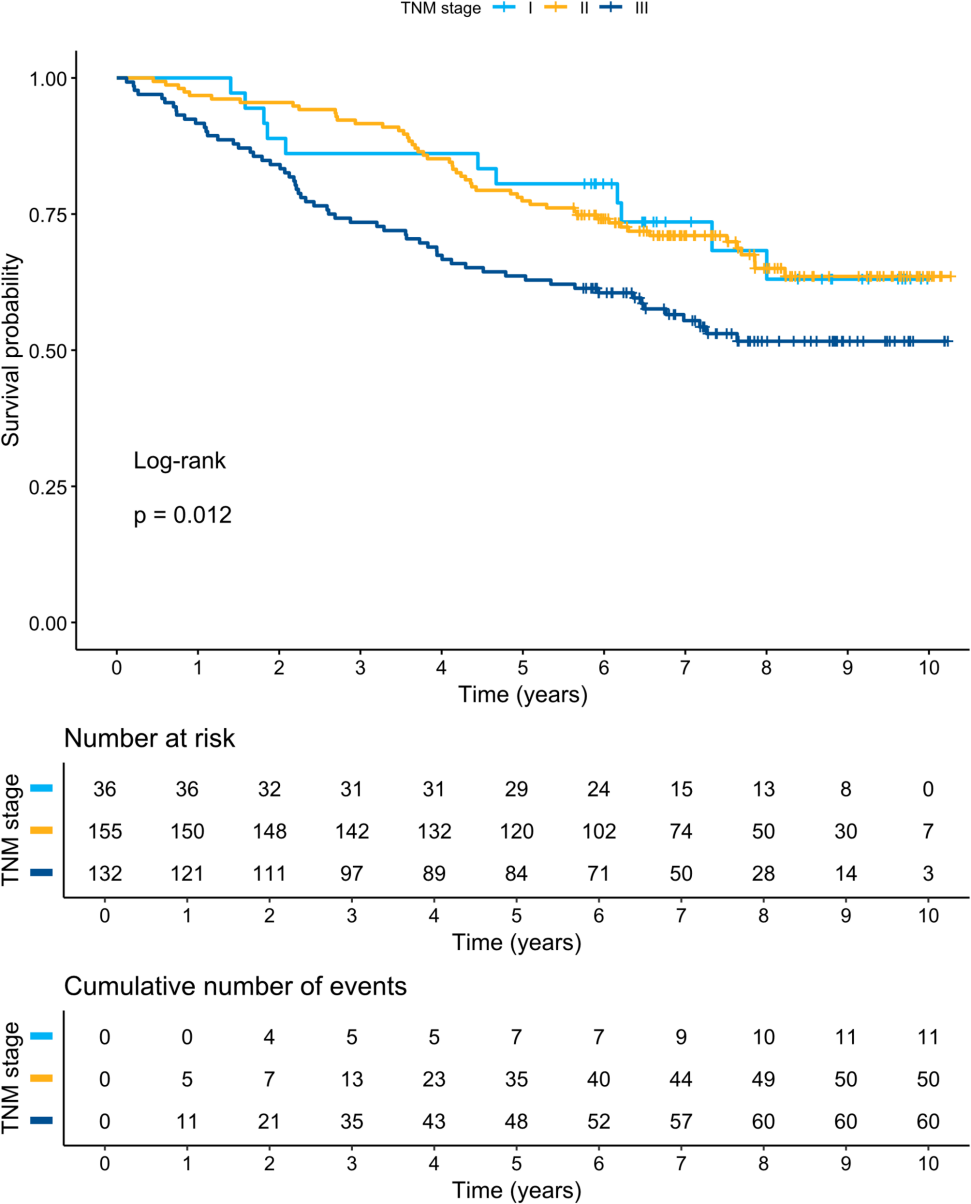
Kaplan-Meier curves for TNM stage in relation to overall survival (OS)

**Fig. 4:**
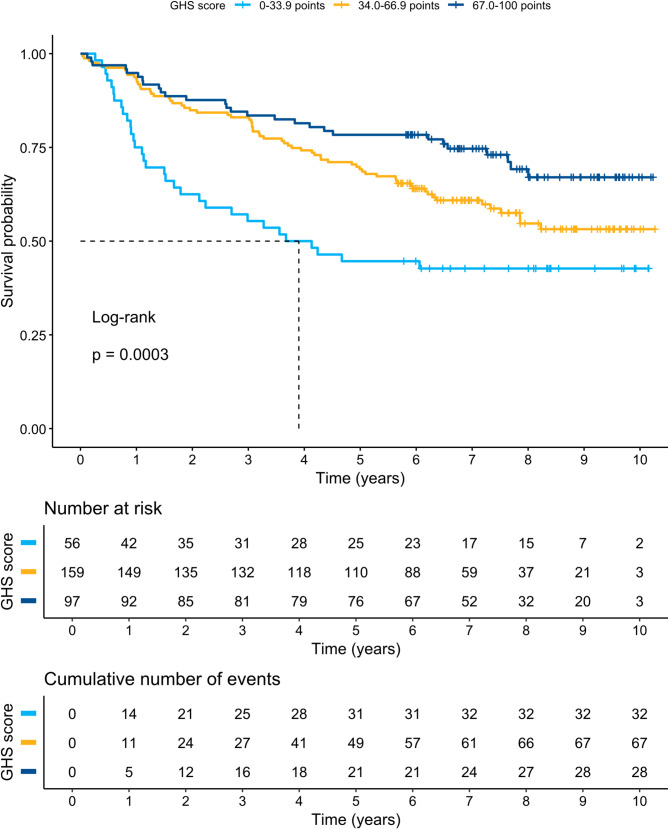
Kaplan-Meier curves for General Health Status (GHS) score in relation to disease-free survival (DFS)

**Fig. 5:**
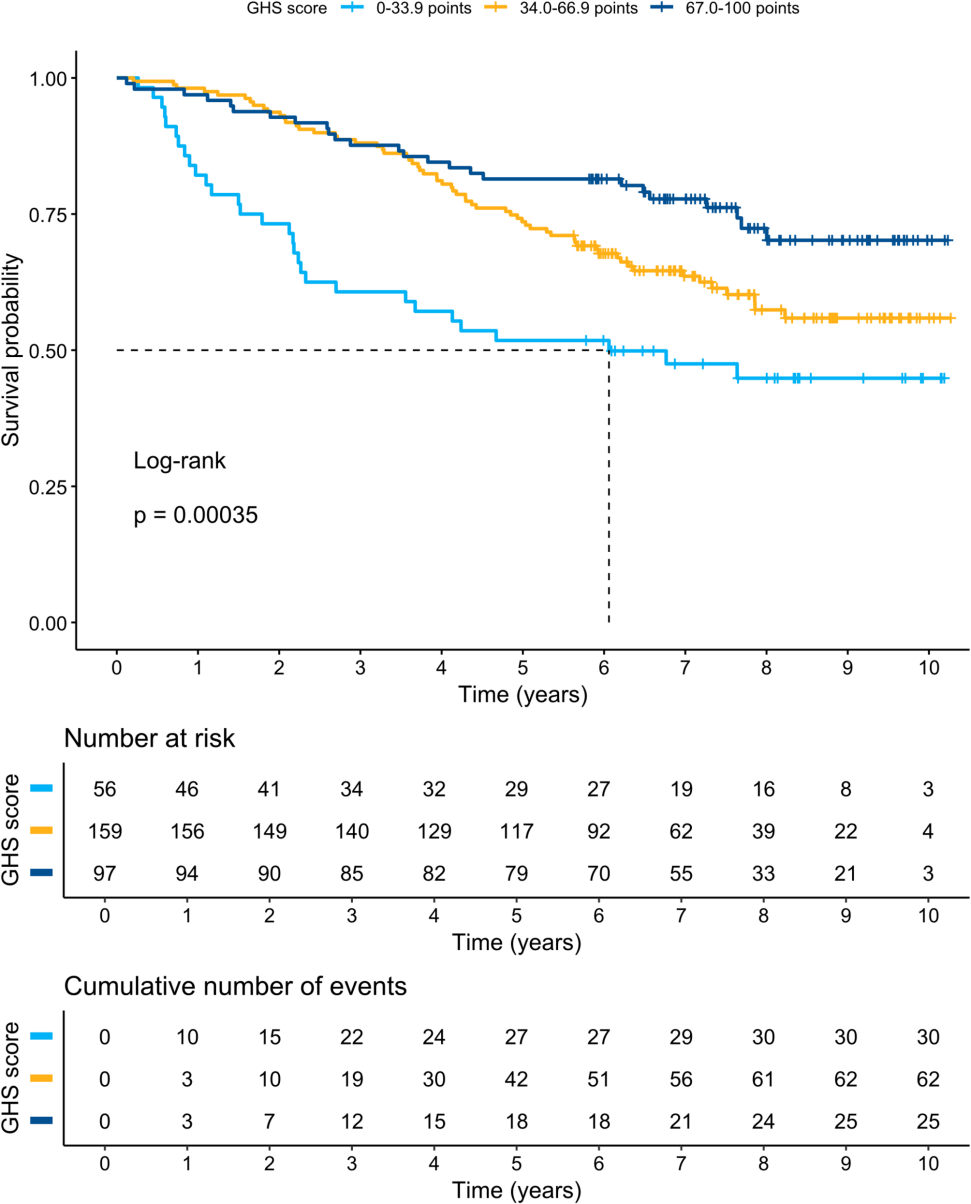
Kaplan-Meier curves for General Health Status (GHS) score in relation to overall survival (OS)

Subgroup analyses of GHS among patients with left-sided, transverse, and right-sided tumours did not show a significant difference in DFS or OS between groups (*p* = 0.16 and *p* = 0.779, respectively) in a Kaplan–Meier log rank (Mantel-Cox) test. Figures 2 and 3 present Kaplan–Meier curves and DFS and OS statistics with different TNM stages (I–III). Figures 4 and 5 present Kaplan–Meier curves and DFS and OS statistics stratified by EORTC QLQ-C30 GHS score, divided into three groups (0–33.9, 34.0–66.9, and 67.0–100 points). According to the log-rank tests, there was a significant difference in DFS and OS among the different groups of TNM stages and GHS scores, as seen in the Figures.

## Discussion

In summary, the results of this study showed that patients with non-metastatic colon cancer (TNM I–III) with a lower GHS, lower physical, role, and social functioning, more fatigue, or more appetite loss at baseline (diagnosis) had both a worse DFS and a worse OS, even after adjusting for established clinical and demographic confounders.

### Results in context

It is well established, but poorly understood, that self-rated health is associated with increased mortality [11, 17]. In heterogenous samples of cancer populations, GHS, physical, social, emotional, and cognitive functioning have been found to be independent prognostic indicators of survival [12, 13]. The question that has remained is how HRQoL can provide insights into predicting cancer recurrence. One hypothesis is that patients who rate their health as poor might relate it to their previous health history and be more aware of their symptoms and mental well-being. An optimistic perception of illness has been shown to be associated with better HRQoL and survival, even if it appears unrealistic with respect to the cancer survivor’s prognosis [18].

Psychological stressors can activate physiological responses that effect antitumour immune function, inflammation, and response to treatment [8, 19, 20]. Chronic stress and a lack of social support have been shown to have an almost ninefold increase in breast cancer incidence [21]. Moreover, a study in patients with breast cancer showed alterations in immunological biomarkers more than one year prior to cancer recurrence [22]. Another study showed that psychological distress and fatigue predicted recurrence and survival in patients with breast cancer [23]. Furthermore, patients who are not feeling well might be less likely to engage in healthy behaviours, which may also increase recurrence risk; however, data on this topic are scarce. The results of this study regarding OS are in accordance with a previous study on patients with colon cancer, in whom HRQoL was measured with the EORTC QLQ-C30 instrument [24]. However, most studies have been conducted in patients with metastatic disease [25, 26] and have shown that pain, appetite loss, and fatigue are the most prominent symptoms [27].

In two recently published papers on patients with non-metastatic colon cancer, self-reported diet, and lifestyle factors [7] and level of distress [10] were shown to predict recurrence. Apart from these studies, to the best of our knowledge, no studies on GHS have been published. There are even less published data on the association between nausea and vomiting and survival in patients with non-metastatic disease, although the present study showed that those symptoms are associated with a worse prognosis. The numeric risk differences were not large, but still significant, and therefore worth presenting, because of their clinical impact and prognostic importance.

### Strengths and limitations

The limitations of this study are that the results were based on patient´s self-reported data, and it was a single-centre study. This study also had several strengths. Firstly, it was population-based. Secondly, HRQoL was measured at baseline, at time of diagnosis, and before treatment, which otherwise could have been a bias. Thirdly, the results were adjusted for established patient- and tumour-related factors. Finally, it should be noted that while self-related health is a valuable predictor of DFS as well as OS, other factors such as genetics, comorbidities not included in the ASA classification, and socioeconomic status are also important predictors of DFS and OS, and the relationship is presumably not casual but can be the result of underlying unmeasured confounders.

## Conclusions

In conclusion, this study showed that assessing HRQoL can be a simple and cost-effective way to identify patients who might benefit from closer medical monitoring and intervention. Thus, it could be an important and valuable tool for more personalised cancer care. Further randomised studies are warranted.

## Data Availability

Data can be sent upon reasonable request assessed by the corresponding author.

## Abbreviations

ASA: American Society of Anesthesiologists
BMI: Body mass index
CI: Confidence interval
DFS: Disease-free survival
EORTC QLQ-C30: European Organisation for Research and Treatment of Cancer Core Quality of Life Questionnaire
GHS: General Health Status
HR: Hazard ratio
HRQoL: Heath-related quality of life
OS: Overall survival
SD: Standard deviation
TNM: Tumour-node-metastasis
